# Psychosocial Assessments for HIV+ African Adolescents: Establishing Construct Validity and Exploring Under-Appreciated Correlates of Adherence

**DOI:** 10.1371/journal.pone.0109302

**Published:** 2014-10-03

**Authors:** Elizabeth D. Lowenthal, Tafireyi C. Marukutira, Jennifer Chapman, Keboletse Mokete, Katherine Riva, Ontibile Tshume, Jessica Eby, Mogomotsi Matshaba, Gabriel M. Anabwani, Robert Gross, Karen Glanz

**Affiliations:** 1 University of Pennsylvania Perelman School of Medicine, Departments of Pediatrics and Epidemiology, Philadelphia, PA, United States of America; 2 Children's Hospital of Philadelphia, Department of General Pediatrics, Philadelphia, PA, United States of America; 3 Botswana-UPenn Partnership, Gaborone, Botswana; 4 Botswana-Baylor Children's Clinical Centre of Excellence, Gaborone, Botswana; 5 University of Pennsylvania Perelman School of Medicine Doris Duke Clinical Research Program, Philadelphia, PA, United States of America; 6 University of Massachusetts Medical School, Worcester, MA, United States of America; 7 Villanova University, Villanova, PA, United States of America; 8 Baylor College of Medicine International Pediatric AIDS Initiative, Houston, TX, United States of America; 9 University of Pennsylvania Perelman School of Medicine, Departments of Medicine and Epidemiology, Philadelphia, PA, United States of America; 10 University of Pennsylvania Perelman School of Medicine and School of Nursing, Departments of Epidemiology and Nursing, Philadelphia, PA, United States of America; Massachusetts General Hospital, United States of America

## Abstract

**Study Objectives:**

Psychosocial factors such as outcome expectancy, perceived stigma, socio-emotional support, consideration of future consequences, and psychological reactance likely influence adolescent adherence to antiretroviral treatments. Culturally-adapted and validated tools for measuring these factors in African adolescents are lacking. We aimed to identify culturally-specific factors of importance to establishing local construct validity in Botswana.

**Methods:**

Using in-depth interviews of 34 HIV+ adolescents, we explored how the psychosocial factors listed above are perceived in this cultural context. We evaluated six scales that have been validated in other contexts. We also probed for additional factors that the adolescents considered important to their HIV medication adherence. Analyses were conducted with an analytic framework approach using NVivo9 software.

**Results:**

While the construct validity of some Western-derived assessment tools was confirmed, other tools were poorly representative of their constructs in this cultural context. Tools chosen to evaluate HIV-related outcome expectancy and perceived stigma were well-understood and relevant to the adolescents. Feedback from the adolescents suggested that tools to measure all other constructs need major modifications to obtain construct validity in Botswana. The scale regarding future consequences was poorly understood and contained several items that lacked relevance for the Batswana adolescents. They thought psychological reactance played an important role in adherence, but did not relate well to many components of the reactance scale. Measurement of socio-emotional support needs to focus on the adolescent-parent relationship, rather than peer-support in this cultural context. Denial of being HIV-infected was an unexpectedly common theme. Ambivalence about taking medicines was also expressed.

**Discussion:**

In-depth interviews of Batswana adolescents confirmed the construct validity of some Western-developed psychosocial assessment tools, but demonstrated limitations in others. Previously underappreciated factors related to HIV medication adherence, such as denial and ambivalence, should be further explored.

## Introduction

More than three million children worldwide are infected with human immunodeficiency virus (HIV),>90% of whom live in sub-Saharan Africa [1]. As the epidemic matures and more children are accessing antiretroviral therapy (ART), children who were born with HIV are aging into adolescence in large numbers [2]. It is estimated that approximately 40% of the more>3 million HIV-infected children worldwide have passed their 10^th^ birthdays [3]. Normative developmental transitions make adolescence a challenging time [4] and adolescents with HIV have worse adherence to treatment than both adults and younger children [5], [6], [7], [8], [9]. Adolescents' adherence challenges endanger not only the wellbeing and longevity of the individual youth, but also threaten to spark a new generation of youth with drug-resistant HIV as HIV+ adolescents with poor treatment adherence engage in sexual activity and potentially transmit resistant virus [10], [11].

When today's adolescents were born, Botswana's HIV epidemic was notable for both the high prevalence of infection and the government's unprecedented proactive response [12], [13]. In 2001, public prenatal clinics in Botswana began to offer free HIV screening and interventions to prevent mother-to-child transmission of HIV (PMTCT). In 2004, the program introduced routine “opt out” testing for HIV, dramatically increasing successful PMTCT efforts among the approximately 37% of pregnant women who were HIV-infected [14]. The government-sponsored treatment program has allowed perinatally HIV-infected children to have successful long-term outcomes [15]. Today, thanks in large part to the successful PMTCT and treatment programs, adolescents comprise the majority of infected pediatric cohorts in Botswana. Countries in the region with less well-developed prevention and treatment programs, however, are similarly seeing increased numbers of perinatally-infected adolescents as their epidemics mature since a substantial minority of perinatally-infected children will survive even without treatment [2].

**Figure 1:**
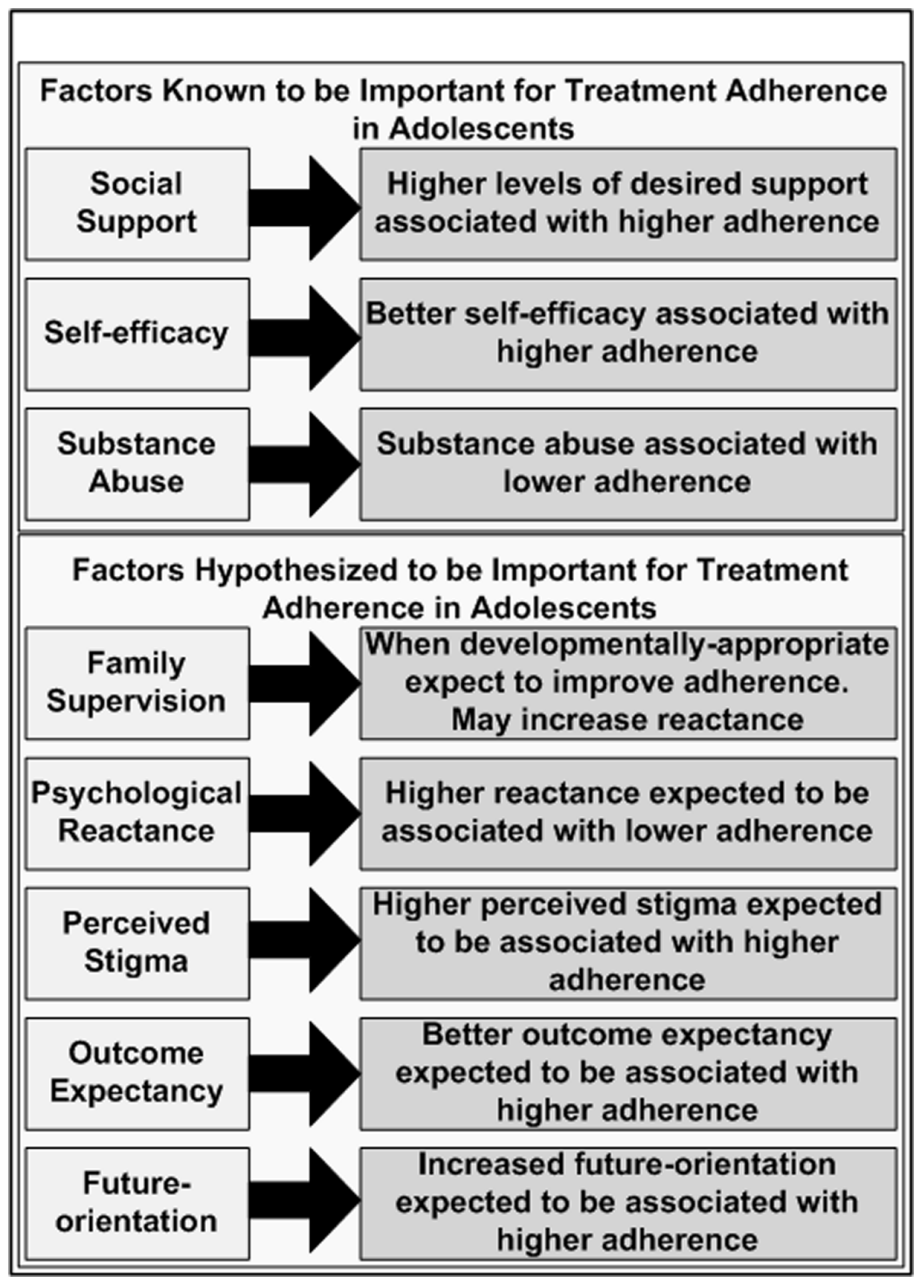
Psychosocial Factors to Be Measured.

The threat of poor adolescent adherence to treatment success and the magnitude of the epidemic among perinatally HIV-infected adolescents have created a substantial public health challenge [16]. Responding to this challenge requires a deeper understanding of the developmental processes and potentially modifiable risk factors for treatment non-adherence among these adolescents. Social support [17], self-efficacy [18] and substance abuse [19], [20] have been shown to be important to adolescent adherence in the U.S., but lack locally-validated measurement tools (See Figure 1). Based on our clinical experience and knowledge of adolescent development, we hypothesized that five separate factors play important roles in making treatment adherence challenging for adolescents. These included family support/supervision, psychological reactance, perceived stigma, outcome expectancy, and future orientation (See Figure 1). Social support can be defined in many ways. Here, we define social support as material and emotional support received from the people whose support is considered most important to the adolescent, as this type of support has been shown to be relevant to adolescent treatment adherence [21], [22]. We define family supervision in a more narrow sense, specifically focusing on the extent to which medication-taking is facilitated and observed by family. Psychological reactance is a strengthening of resistance to a desirable action in response to perceived threats to behavioral freedom. It peaks during adolescence and leads to increases in risk-taking behaviors [23].

Tools to measure these factors have similarly not been culturally-adapted and validated for use among adolescents in Botswana. We aimed to culturally-adapt and establish the construct validity of tools designed to measure these constructs. Construct validity is the extent to which a test measures what it claims to measure [24]. A measure that accurately measures a factor of interest in one culture may not do so in another cultural context [25].

The tools selected for adaptation have been validated in other settings, but not among adolescents in sub-Saharan Africa. They included the Hong Psychological Reactance Scale [26], The Stigma Scale Revised [27] (perceived stigma), the Antiretroviral Medication Attitudes Scale [28] (outcome expectancy), the Consideration of Future Consequences Scale [29], the Child and Adolescent Social Support Scale (CASSS) [30], [31], [32], the HIV Medication Self-efficacy Scale [33], and the World Health Organization's Smoking and Substance Involvement Screening Test (ASSIST) [34], [35]. For family supervision, no existing tool was found that described the types of supervision received by our patients. So, it was decided that for this construct, a simple tool would be created *de novo* based on adolescents' descriptions of the type of supervision and support they receive when taking medication.

In this study we utilized in-depth interviews with adolescents to establish the construct validity of some Western-developed psychosocial assessment tools while demonstrating limitations in others. We describe the process of culturally-adapting the measurement tools and highlight data justifying decisions to alter aspects of some tools for local use in Botswana. The culturally-adapted tools which required more than minor changes are included in Appendix S1.

## Methods

Adolescents were chosen for study enrollment from among the 1472 10–19 year olds who were receiving antiretroviral therapy at the Botswana-Baylor Children's Clinical Centre of Excellence in Gaborone, Botswana. A stratified sampling approach was employed to enroll a minimum of 10 adolescents (5 males and 5 females) in each of 3 age-bands (16–19 years, 13-<16 years, and 10-<13 years). On days when the study was enrolling, adolescents in each enrolling stratum were sequentially approached for enrollment when they presented for routine clinic visits. Enrollment was continued beyond the minimum number of enrollees until thematic saturation was achieved. To be eligible for enrollment, all adolescents had to be aware of their HIV status and had to be prescribed antiretroviral therapy. For>90% of patients in this clinical setting, the mode of HIV acquisition is thought to have been perinatal.

Interviews were conducted in either English or Setswana, whichever language was preferred by the adolescent. The interview guides were translated into Setswana by a single study team member and the translations were subsequently reviewed and improved by all bilingual study team members as well as additional clinic staff. Interviews were conducted by two research team members who were trained to perform qualitative research. Interviews were conducted in a private location within the clinic where the adolescents receive their HIV care. For some of the interview sessions, both interviewers were present. No other observers were present during interviews. Interview sessions were audio-recorded, transcribed and translated into English (if needed). Interviews were transcribed and translated by the same individual who conducted the interview. Following transcription and translation, the audio recordings and interview transcript(s) were reviewed by at least one additional team member to ensure accuracy. The study Principal Investigator subsequently reviewed all transcripts and requested clarifications and alternative translations when aspects of the transcript were unclear.

Interviews were conducted in 3 phases, beginning with the oldest adolescents (age 16–19 years). The interview guide for the oldest adolescents focused on gaining a broad understanding of what the adolescents thought of each of the 5 constructs of interest outlined in Figure 1. It began with broad questions such as “What does stigma mean to you?” Follow-up questions aimed to elicit conversation about how each of the constructs operates in their lives. (e.g., “Some people with HIV fear that people will treat them differently if they know that they have HIV. What are things that people in Botswana sometimes say and do to treat people with HIV differently?”). The concept of psychological reactance was framed for the adolescents using two vignettes, one involving an obedient teenager and the other involving a reactant teenager. In both vignettes, the adolescent is told by her parent that she could not go to a party in the city with new friends. In the obedient vignette, the teen stated that she was not sure that she wanted to go to the party. Since her father did not want her to go, she simply made other plans with different friends. In the reactant vignette, although the adolescent was not sure that she wanted to go the party, she was so angry that her father told her that she could not go that she climbed out of her bedroom window to “sneak out” to the party. The adolescents were asked to describe instances in which they have seen peers respond in ways that similarly demonstrated reactance.

Additional questions were asked to address the appropriateness of the scales chosen to measure social support and substance use. The Child and Adolescent Social Support Scale measures perceived social support from many sources. In order to determine the extent to which measuring these different domains would be important in our cohort, we asked the adolescents to rate on a scale of 1 to 10 how important support was from parents, close friends, teachers, community leaders, church members, and other family members. They were then asked to indicate why they chose their ratings.

Interviews for the middle age group (age 13-<16 years) focused on exploring areas in which the interviews with the oldest group suggested the need for additional clarification. Like the interviews with the oldest adolescents, these utilized primarily open-ended questions and related to the factors indicated in Figure 1. Questions for which thematic saturation was achieved during the interviews with the oldest adolescents were excluded from the interviews with the middle age group. The interviews with the middle adolescents added further exploration of medication-taking autonomy development, parental definitions, and exploring adolescents' goals for the future.

Coding summaries were reviewed by the entire study team between the interviews of the oldest and middle groups and the interviews of the middle and youngest groups. Following the middle age group review, the team adapted the tools based on the adolescents' feedback. Interviews with the youngest adolescents followed the tool adaptation. Because of the developmental limitations of most 10–13 year olds, we used more concrete questioning for this group. The majority of the time spent with the youngest adolescents was spent reviewing each of the survey questions. The interviewer asked the young adolescents to discuss how easy it was to understand each question and whether alternate wording should be considered.

All adolescents in each of the three interview phases were asked to inform the interviewers of anything important to them and their HIV+ peers that was excluded from the interview questions. Each interview took between 30 and 60 minutes. Once transcripts were finalized, they were coded using an analytic framework approach with NVivo9 software [36]. Each script was coded by the study PI and one of two additional team members. We took an analytic framework approach whereby codes were organized into categories relevant to the constructs of interest and additional categories were suggested for themes that arose from the interview data [37]. After each transcript was coded by two or more researchers, discrepancies were reviewed and resolved.

Demographic data were collected and managed using REDCap electronic data capture hosted at Children's Hospital of Philadelphia [38]. Quantitative analyses of demographic data and social support importance rankings were conducted using Stata/IC 11 (STATA Corp. College Station, TX) [39].

### Ethics Statement

Approvals for the conduct of this study were obtained from the research ethics boards of the Botswana Health Research Development Council, the University of Pennsylvania, and Baylor College of Medicine. Written informed consent was obtained from the parent or guardian and written assent was obtained from all study subjects. All anonymous study scripts can be made freely available for further scientific inquiry by written request and agreement with the Principal Investigator.

## Results

The study population consisted of 18 females (53%) and 16 males, with a median time on antiretroviral therapy of 84 months (IQR 60–120 months, range 0.5–216 months). Twenty adolescents (59%) chose to complete the in-depth interviews in English. Of all eligible adolescents approached for study inclusion, only two were excluded (due to lack of parental consent).

### Relevance and Understandability of Concepts and Questions

Some Western tools were well-understood and relevant to Batswana youth, requiring only minimal modifications (please see Scale Modifications section below for details). These included the Stigma Scale Revised and the Antiretroviral Medication Attitudes Scale. The ASSIST tool was well-understood with the inclusion of local terms for drugs of abuse. All of the other tools lacked some cultural relevance and/or were in part difficult for Batswana youth to understand. Table 1 presents representative quotations illustrating how the adolescents described the locally-important adherence barriers not represented in the tools. When no interviewer question is listed in the table, the participants provided the information as part of the response to a more exploratory question. Most questions included in the HIV Medication Self-efficacy Scale were relevant to the adolescents. However, absent from the tool were situations the adolescents highlighted as most challenging for maintaining medication adherence. Traveling, particularly school trips, were highlighted. The adolescents also referred to fear of loss of confidentiality when taking antiretrovirals in front of people unaware of their HIV status, such as in the company of friends, visitors in the homes, or visiting relatives' homes.

**Table 1:** Quotations Illustrating Key Themes.

Construct	Representative Quotes	
Self-efficacy	Interviewer: Can you think about times when it is difficult for you or other people your age to take their medicines?	*… when they go for a school trip because most of the bags, they stay at one side. So, when you go and take your pills … your friends … always ask “where are you going?” And it's difficult to tell them* (17 year old female, on treatment for 8 years.)
		*Well, sometimes it's hard when you are with friends chilling.* (17 year old male, on treatment for 7 years)
		*Every Saturday we go to the lands [where we grow crops] and we come in the evening. So, sometimes we come late for the [medication] time.* (18 year old male, on treatment for 13 years)
		*… when schools close … someone will want to take you for a week and maybe that person doesn't know that you are taking medication and you end up not telling him or her because you think he or she might end up hating you because of the virus …. (16 year old female, on treatment for 10 years)*
Future	Marriage	*A person who is HIV negative will just marry properly, but the one who is HIV positive would be scared, like how am I going to tell her one day.* (15 year old male, on treatment for 6 years)
		*If I'm going to get married…I have to let my husband know that I am HIV positive and that is going to be a hard thing for me to do.* (15 year old female, on treatment for 3 years)
	Children	*I am just going to adopt because I am afraid of having sex with a girl because that will give me more diseases.* (16 year old male, on treatment for 7 years)
		*I fear that I may have children and not be able to take good care of them… (because of my HIV status).* (14 year old female, on treatment for 2 years)
	Employment and Supporting Families	*I think of the time I will work, buying food, paying electricity and water bills.* (14 year old female, on treatment for 13 years)
		*Because for a person like me who is HIV positive and a friend of mine who is HIV negative, when I say I want to be a doctor she also has the same plan.* (14 year old female, on treatment for 3 years)
		*In the future I will build (my family) a big house just to thank them for having brought me up.* (14 year old female, on treatment for 2 years)
	Adult Health	*Those infected will be thinking I may get sick and don't have the future that I want. Those who are negative will think of the future without worrying that they are sick.* (14 year old female, on treatment for 2 years)
		*Since there is no cure, … I think that I'm not going to reach or manage so many years ….* (17 year old male, on treatment for 7 years)
		*They think of their future a lot more than the present…the HIV positive. They think about …if they will be well and if they will be here someday.* (014, 14 year old female, on treatment for 6 years)
Psychological Reactance	*I feel like she treats me like a, a little child, you know? Even though I am big enough to remember. …[If my mom reminds me when I'm not in a good mood], I tend to leave (the medicines)* (17 year old male, on treatment for 7 years)	
	*It depends in the mood I am in. If I am in the bad mood, it will be like I am really forced to do something I don't want to do. If you are doing something for the sake of being healthy and somebody talks about it too much it makes me sick.* (16 year old female, on treatment for 10 years)	
	*When my mom asked me to drink the pills I feel angry and then I opened the bottle and then take the pills then close and then don't (consume them). I pretend. …When it's my time to drink the pills, I drink when you are not asking me to drink.* (17 year old female, on treatment for 8 years)	
Parental Figures	Interviewer: *When you talk about your parents, who exactly do you mean?*	*I mean mum, uncles, cousins, brothers and my aunts.* (14 year old female, on treatment for 2 years)
		*My grandmother, I can call her mum and grandfather, I can call him dad.* (16 year old male, on treatment for 7 years)
		*Mum is the only one because she raised me* (15 year old male, on treatment for 8 years)
Perceived Social Support	Parents	*(Support from parents) is very, very, very important because if they abandon you then just think that it's your family who is supposed to be helping you…. That's the reason why some people commit suicide, because they lack support from parents.* (16 year old female, on treatment for 10 years)
		*They are the most important supervisors you stay with. They are also very close to you. You can express your feeling to them…very, very important.* (18 year old male, on treatment for 10 years)
	Friends	*I think (support from a friend is) important but not that important because…even though it's a close friend you don't have to…disclose your status. Some people find it difficult to tell a friend because they don't know whether the friend will last or what.* (17 year old female, on treatment for 8 years)
		*… you might be close but they change every time. They are not so close like your parents, your family, so it can come out to be your enemy sometimes and expose you.* (18 year old male, on treatment for 10 years)
		*It's important for your closest friends to help you so that you don't feel abandoned, yah! But it is not that very, very important. Like if you have the family support, yeah, I think you have all the support you need.* (16 year old female, on treatment for 10 years)
	Teachers	*For teachers, I don't think it's important because they are…not close to us. It's just that we spend a year with them… I don't think it's important.* (17 year old male, on treatment for 7 years)
	Community leaders	*Children nowadays are into celebrities, not community leaders.* (18 year old male, on treatment for 8 years)

Two questions from the Consideration of Future Consequences Scale were considered both relevant and understandable. These included: “I believe that I am in control of what will happen to me in the future” and “I am willing to sacrifice my immediate happiness for things that I expect to happen in the future”. However, the original scale includes abstract concepts about the future that did not resonate with the adolescents despite attempts at explanation. The adolescents expressed concrete concerns about their futures, particularly their ability to marry, have children, obtain employment, support their families, and maintain health as adults.

The Hong Psychological Reactance Scale was not intended to measure psychological reactance related specifically to medication adherence. Yet, the youth noted that adolescents might act rebellious when reminded to take their HIV medications, even if they would not be rebellious in other contexts such as that described for the subject of the vignette. Thus, the concept of reactance was clearly understood. However, the Hong Psychological Reactance Scale also contains jargon such as “acting of my own free will” that was difficult for the adolescents to comprehend.

The adolescents consistently expressed that social support from parental figures is of the greatest importance to them. On a scale of 0–10 with 10 being the most important, 100% indicated that support from parents was 10/10 or “very important”. Most also considered support from other family members to be very important, median (interquartile range (IQR)) score 10 (7–10). In contrast, the median (IQR) score for support from close friends was 5 (5–6). Support from teachers, community leaders and church members were ranked as being of only moderate importance to most of the adolescents with median (IQR) scores of 5 (4–5), 5 (2–5), 4.5 (3–7), respectively. The adolescents referred to a number of relatives as “parents”, with “parental relatives” being those who played the most prominent roles in their upbringing and with whom they felt the closest. “Parents” were described as those who provide for them both emotionally and materially.

### Unsolicited Themes: Denial and Ambivalence

Although not solicited directly, the adolescents expressed that issues of denial and ambivalence toward HIV are important to them and their peers. In some subjects, having been without illness for long periods of time seemed to result in a sense of denial about their HIV status. Some noted that medication non-adherence made them feel physically better with the resolution of side effects. This complicated their ability to maintain a sense that the treatment increases their wellness. Some of the most salient quotes related to denial and ambivalence are outlined in Table 2.

**Table 2:** Denial and Ambivalence Statements from Adolescents.

Interviewer: *What are some attitudes that you think people your age with HIV have regarding their medicines?*	*…like right now, I am thinking…I know that I am healthy. They will think that…they are healthy and they will not take their medicines.* (18 year old male, on treatment for 13 years)
	*…they don't think they are HIV positive. And they think they are fine because …there are no signs that show me that I'm sick, that I'm not healthy. Sometimes I don't drink [the medicines] for a month.* (17 year old female, on treatment for 8 years)
	*… Sometimes it's hard to appreciate that you have the virus and you don't believe until maybe something has happened to you. … Apparently a year back, I was totally gone. I truly believed I was dead this time. That's when I truly realized there is no any other way. … For the teens, it's very difficult to appreciate they have HIV and take medicine well unless if something has happened to them.* (18 year old male, on treatment for 10 years)
Interviewer: *Why do you think that medications don't improve quality of life for people your age?*	*Because they don't believe that they are HIV positive…right now I am not accepting, I'm not believing that I'm HIV positive.* (17 year old female, on treatment for 8 years)
Interviewer: *What else can you think of that might make it difficult for you or other people your age to take their medications?*	*Some people at the beginning…they did not accept their status…that they are HIV positive.* (18 year old female, on treatment for 5 years)
	*I think people are in denial and they know they should take their medicines religiously but they don't…because they believe that it will just go away but in reality it won't.* (17 year old female, on treatment for <1 year)
Interviewer: *Do you consider yourself to be healthy?*	*It's like I have two “hearts”, the other one tells me that “you are healthy. Don't drink the pills. You are healthy, no pains, nothing”. This I am saying from my past experience. The other one says “you should drink the pills because you are not healthy; mogare [virus] is awake in your body”. Then I will just leave the pills because I believed I was healthy.*(15 year old male, on treatment for 8 years)

### Scale Modifications

Two questions on the Stigma Scale Revised were relevant to only a subset of the patients. These ask if the individual has “been hurt by how people reacted to learning (they) have HIV” and if the individual has “lost friends by telling them that (they) have HIV”. Since many adolescents do not disclose their HIV status to anyone, our revised scale first asks if the adolescent has ever told anyone that he or she has HIV. Only if that question is answered in the affirmative will the two disclosure-related questions be asked. Four items on the 14-question Antiretroviral Medication Attitudes Scale that were considered less relevant, redundant or confusing to our adolescents were removed [28]. These included the following questions: 2- “My defenses are stronger on my medication”, 5- “I will try any medication if I think it will help me to live longer”, 10- “Things that I could do easily are much more difficult when I am on my medication”, and 13- “Taking my medication will prevent me from being hospitalized”.

The HIV Medication Taking Self-efficacy Scale (self-efficacy subscale) asks about specific situations in which patients sometimes struggle to take their medications. We removed some scenarios (i.e. “at a planned event”, “at an unplanned event”, “at a party”, “at a social outing”, “at work”) that were considered of limited relevance or redundant to our adolescents. We added questions about taking medications when on school trips and when people are visiting the home as these were highlighted as being frequently problematic situations for our youth.

A new reactance scale (The “Botswana Adolescent Medication-related Reactance Scale”) was created that utilized 3 salient questions based on concepts from the Hong Psychological Reactance Scale and 2 medication-specific reactance questions. These include:

1. I do not like to follow rules.
2. When something is against the rules, I usually think, “That's exactly what I am going to do”.
3. I become irritated when I am unable to make decisions for myself.
4. When someone tells me to take my pills, it makes me want to avoid them.
5. I get angry when I am reminded to take my pills.

Similarly, because the participating adolescents did not relate well to the Consideration of Future Consequences Scale, we created a new 5-question scale (The “Botswana Adolescent Beliefs about the Future Scale”) based on what the adolescents told us is of concern to them and their peers related to the future. The scale does include two more general questions about the future that are based on concepts from the Consideration of Future Consequences Scale that our adolescents understood and considered relevant: “I am willing to sacrifice my immediate happiness for things that I expect to happen in the future” and “I believe that I am in control of what will happen to me in the future”. The three questions regarding goals for the future include:

1. When I am an adult, I think I can have my own children if I want to.
2. When I am an adult, I think I will be able to get a job that I like.
3. I believe that if I take my medications, I can grow to be a healthy adult.

We sought to shorten the Child and Adolescent Social Support Scale, which asks about the types of social support received from many different sources. Because of the adolescents' statements about the types of support that they receive and its importance, our version contains only two sections. The first asks about support received from parents. The second asks if there is anyone who helps the adolescent as much as his or her parents. If they answer “yes” to this question, the support from that individual is also assessed. For defining parental supervision of medication-taking, we designed a single multiple-choice question to help distinguish between the levels of supervision and autonomy that were reported:

Which statement best describes how you take your medicines on most days:

- On most days I take my medicines by myself with nobody reminding me or watching me.
- On most days I take my medicines by myself, but someone reminds me to take them.
- On most days, somebody gives me the medicines when it is time for me to take them, but I take them by myself.
- On most days, somebody gives me the medicines and watches me swallow them.

A two-question denial scale was created to address the concerns that the adolescents raised about denial and ambivalence. The scale allows the adolescents to indicate whether they do not want to take their medicines because they think they are too healthy “never”, “some of the time”, or “all of the time”. Using the same scale, it asks “I do not believe that I have HIV”.

The answer scales for the other tools were harmonized to make them less confusing for adolescents answering these questionnaires together as part of an assessment battery. The original versions of the Hong Psychological Reactance Scale and the Stigma Scale Revised use a 5-point scale from “strongly disagree” to “strongly agree”. The Antiretroviral Medication Attitudes Scale uses a 5-point scale from “definitely false” to “definitely true”. The Consideration of Future Consequences Scale uses a 5-point response scale from “very uncharacteristic of me” to “very characteristic of me”. The HIV Medication Taking Self-Efficacy Scale uses a 10-point response scale from “not confident” to “totally confident”. In our revised testing materials, we use the 5-point scale from “definitely false” to “definitely true” for reactance, stigma, outcome expectancy, and beliefs about the future. For the Botswana version of the HIV Medication-Taking Self-Efficacy Scale, we use a 5-point scale from “not at all confident” to “totally confident”.

## Discussion

We studied issues of importance regarding adherence to ART among adolescents who have grown up with HIV in a high prevalence setting in order to culturally-adapt relevant assessment tools. We found that some existing tools for measuring psychosocial variables potentially associated with treatment adherence had good construct validity for adolescents in Botswana. Specifically, the Stigma Scale Revised and the Antiretroviral Medication Attitudes Scale were deemed relevant and well-understood. However, other tools that have been previously validated for different populations were found to be lacking for adolescents in our setting. For example, the HIV Medication Self-efficacy Scale failed to include situations in which our adolescents said that their medication self-efficacy is most challenged. The Consideration of Future Consequences Scale did not capture concrete concerns about the future that the adolescents verbalized as relating directly to their ability to maintain their health. The need for medication-specific psychological reactance questions was also highlighted.

Although we know that family plays a more important lifelong role in decision-making in this cultural context than in many Western contexts [40], we were surprised that adolescents in our study ranked the personal importance of peer support well-below that of family and more on par with that of teachers, community leaders and church members. Numerous prior studies stress the importance of peer relationships, support and acceptance to adolescent development and decision-making [41], [42], [43]. Adolescents spend more of their waking hours with peers than with adults [44]. Prior studies in Botswana have confirmed the local importance of peers to some aspects of adolescent decision-making [45]. Since adolescents in our study suggested that peer support is significantly less important than family support, our simplified social support assessment scale allows adolescents to identify a peer, a family member, or any other individual as an important supporter rather than focusing specifically on peer-support.

Because of developmental differences, the same tools may not be valid across the entire age range of 10–19 year olds. One of the strengths of this study was the inclusion of stratified sampling to help ensure that the concepts were both salient and comprehensible across the adolescent developmental spectrum. Other strengths included continuation of interviews until thematic saturation was achieved and the use of a multicultural and multidisciplinary team to analyze and interpret results. The adolescents were sampled from a large clinic which serves children and adolescents from both urban and rural settings. The main limitation of the study is that it did not go beyond cultural adaptation and construct validation to assess other aspects of reliability and validity which require different methods and larger sample sizes. By changing the self-efficacy scale from a 10-point scale to a 5-point scale, we sacrifice granularity and likely will achieve a slightly higher mean score than would be expected with a 10-point scale [46].

These findings stress the need for cultural adaptation of tools that were designed for use in other contexts prior to their use in settings such as Botswana. Unfortunately, existing tools to measure issues of likely importance to adolescent treatment adherence have been developed almost exclusively in low HIV-prevalence settings such as the United States. Cross-cultural adaptation of psychosocial assessment measures is an essential step to ensuring the quality of data obtained using those measures in new cultural contexts [47]. Only when the quality of the cultural adaptation and translation process has been reported can researchers draw appropriate conclusions about constructs assessed and differences between studies and datasets [48]. There is a growing body of literature suggesting that a simple translation and back-translation process is insufficient to ensure the appropriateness of a new test version [49], [50], [51]. Our own prior experience in Botswana suggested that using a multidisciplinary team of skilled bilingual professionals followed by limited pilot testing may fail to produce a culturally-appropriate adaptation of complex tools to measure constructs such as depression and anxiety symptoms [52].

The goal of scale revision is to improve the validity of measures. Next steps for this work will include assessing the internal consistency and test-retest reliability of these tools in a population of HIV-infected adolescents who were not included in this qualitative study. Other techniques such as factor analysis and multidimensional scaling may also be employed to clarify the cross-cultural validity of tools [53], [54]. If deemed to be valid and reliable, the tools will be used to longitudinally assess the relationships between these psychosocial measures and adherence to antiretroviral therapies in adolescents in Botswana. The psychosocial assessment tools developed for use among HIV+ adolescents in Botswana might require minimal adaptation for use in similar settings. Researchers and clinicians seeking to use these tools among HIV-infected adolescents outside of Botswana should consider differences in language and culture as well as HIV prevalence and treatment access in making decisions regarding the need for re-adaptation for their settings. We recommend that researchers seeking to quantify psychosocial constructs among populations in settings where validated locally developed tools do not exist, utilize qualitative methods such as those described in this paper to ensure cultural appropriateness, construct validity and comprehensibility of the measures.

## Conclusions

In-depth interviews of HIV-infected adolescents in Botswana confirmed the construct validity of some Western-developed psychosocial assessment tools that could be used to assess the constructs' relationship to treatment adherence in this and potentially similar populations. Denial and ambivalence emerged as underappreciated factors related to HIV medication adherence that need to be explored further.

## Supporting Information

Appendix S1 (DOCX)
