# Supplementary material for: Psychosocial Assessments for HIV+ African Adolescents: Establishing Construct Validity and Exploring Under-Appreciated Correlates of Adherence
Source: PLoS One. 2014 Oct 3;9(10):e109302. doi: 10.1371/journal.pone.0109302 (PMC4184864; doi:10.1371/journal.pone.0109302)
Supplement: Appendix S1 — (DOCX) [file pone.0109302.s001.docx]

**Botswana Adolescent HIV Denial Scale**

1 2 3

Some of the Time

Nako tse dingwe

Never

Gotlhelele Ganke

All of the Time

Nako tsotlhe

1. I do not want to take my medicines because I think I am too healthy.

Ga ke batle go tsaya/nwa melemo yame ka gore ke akanya gore ke itekenetse tota.

1. I do not believe that I have HIV.

Ga ke dumele gore ke nale HIV.

**Botswana Adolescent Beliefs about the Future Scale**

Please answer the following questions with this scale of 1 to 5.

Tswee tswee araba dipotso tse di latelang ka sekale sa go tswa mo bongweng go ya ko botlhanong.

**1 2 3 4 5**

Definitely True

Ke nnete tota

Somewhat True

Ke nnete

Not True or False

Ke fa gare

Somewhat False

Ga se nnete

Definitely False

Ga se nnete tota

1. I am willing to sacrifice my immediate happiness for things that I expect to happen in the future.

Ke eletsa go ntsha setlhabelo tse di intumedisang ka nakwana gore ke kgone go fitlhelela maduo a bokamoso.

1. I believe that I am in control of what will happen to me in the future.

Ke dumela gore ke mo taolong ya se se tla ntiragalelang mo bokamosong.

1. When I am an adult, I think that I can have my own children if I want to.

Fa kele mogolo,ke akanya gore ke ka nna le bana ba e leng bame fa ke batla.

1. When I am an adult, I think that I will be able to get a job that I like.

Fa kele mogolo,ke akanya gore ke tla a kgona go bona tiro e ke e ratang.

1. I believe that if I take my medications, I can grow to be a healthy adult.

Ke dumela gore fa ke nwa melemo yame, ke tla a gola go nna mogolo yo o itekanetseng.

**Botswana Adolescent Medication-related Reactance Scale**

Please answer the following questions with this scale of 1 to 5.

Tswee tswee araba dipotso tse di latelang ka sekale sa go tswa mo bongweng go ya ko botlhanong.

**1 2 3 4 5**

Definitely True

Ke nnete tota

Somewhat True

Ke nnete

Not True or False

Ke fa gare

Somewhat False

Ga se nnete

Definitely False

Ga se nnete tota

| 1. I do not like to follow rules.   Ga ke rate go sala morago melao. |
| --- |
| 1. When something is against the rules, I usually think, “That’s exactly what I am going to do”.   Fa sengwe se se kafa molaong ,ke nna ke akanya,"ke sone se tota se ke yang go se dira" |
| 1. I become irritated when I am unable to make decisions for myself.   Ke a tenega fa ke sa kgone go itseela ditshwetso ka bo nna. |

1. When someone tells me to take my pills, it makes me want to avoid them.

Fa mongwe a mpolelela gore ke nwe dipilisi tsa me, go ntira gore ke leke go di ikgatholosa.

1. I get angry when I am reminded to take my pills.

Ke a tenega fa ke gakololwa gore ke nwe dipilisi tsame.

**HIV Medication-Taking Self-Efficacy Scale- Botswana Version**

Please answer the following questions with this scale of 1 to 5.

Tswee tswee araba dipotso tse di latelang ka sekale sa go tswa mo bongweng go ya ko botlhanong.

**1 2 3 4 5**

Not At All Confident

Ga ke tlhomamise tota

Totally Confident

Ke a tlhomamisa tota

Very Confident

Ke a tlhomamisa

Moderately Confident

Ke fa gare

Somewhat Confident

Ga ke tlhomamise

1. How confident are you that you can take your medication at the right time every day?

O tlhomamisa go le kana kang gore o ka tsaya/nwa melemo ya gago ka nako malatsi otlhe?

1. How confident are you that you can take the correct amount of medicine every time?

O tlhomamisa gole kana kang gore o tla a tsaya/nwa se lekanyo se se tshwanetseng nako tsotlhe?

1. How confident are you that you can take your medicines on weekends (Saturday and Sunday)?

O tlhomamisa go le kana kang gore o ka tsaya/nwa melemo ka mafelo a beke? (ka matlhatso le tshipi)?

1. How confident are you that you can take your medicines on weekdays (days other than Saturday and Sunday)?

O tlhomamisa gole kana kang gore o ka tsaya/nwa melemo ya gago fagare ga beke?

(malatsi a mangwe kontle ga matlhatso le tshipi)?

1. How confident are you that you can take your medicines at a party or other social event?

O tlhomamisa go le kana kang gore o ka tsaya/nwa melemo ko meketeng kana bokopano bope fela?

1. How confident are you that you can take your medicines when there are people visiting your home?

O tlhomamisa gole kana kang gore o ka tsaya/nwa melemo ya gago fa go nale batho ba ba etetseng ko ga lona?

1. How confident are you that you can take your medicines when you are traveling away from home?

O tlhomamisa go le kana kang gore o ka tsaya/nwa melemo fa o le mo mosepeleng kgakala le legae?

1. How confident are you that you can take your medicines when you are not sick?

O tlhomamisa go le kana kang gore o ka tsaya /nwa melemo ya gago fa o ikutlwa o ka re ga o lwale?

1. How confident are you that you can take your medicines when you feel ill?

O tlhomamisa gole kana kang gore o ka tsaya/nwa melemo ya gago fa o ikutlwa o lwala?

1. How confident are you that you can take your medicines when you are having medication side effects? (An example of a medication side effect is feeling like the medicines gave you a stomach ache.)

O tlhomamisa gole kana kang gore o ka tsaya/nwa melemo ya gago fa o nale ditlamorago tsa melemo? (sekai sa ditlamorago ke go ikutlwa e kare molemo o dira gore mpa e nne le ditlhabi)

1. How confident are you that you can take your medicines when you are on school trips?

O tlhomamisa gole kana kang gore o ka tsaya/nwa melemo ya gago o le mo loetong la sekole?

1. How confident are you that you can take your medicines when there are difficult things happening in your life?

O tlhomamisa go le kana kang gore o ka tsaya/nwa melemo ya gago fa gona le dilo tse di thata tse di diragalang mo botshelong jwa gago?

**Botswana 2-module Child and Adolescent Social Support Scale**

I am going to ask you about different kinds of help that you get from your parents. For each kind of help, I want you to tell me two things. First, tell me how often you receive the kind of help described. Then, rate how important you think that help is to you. For the part when you tell me how often you get that type of help, we are going to use the following choices:

Ke tlile go go botsa ka mefuta e e farologanyeng ya dithuso tse o di amogelang go tswa mo batsading. Mo mofuteng mongwe le mongwe wa thuso, ke batla o mpolelele dilo dile pedi. Lantlha, mpolelele gore o amogela mofuta wa thuso o o tlhatositsweng ga kae. O bo o tshwaya gore o akanya thuso e o, ele botlhokwa go le kana kang mo go wena. Mo karolong e o mpolelelang gore o bona mofuta wa thuso e o ga kae re tlile go dirisa tse di latelang:

**1 2 3 4 5**

Never

Gotlhelele Ganke

Almost Always

Gotlhelele nako tsotlhe

Always

Nako tsotlhe

Some of the Time

Nako tse dingwe

Almost Never

Ganke

For the part where you say how important the help is, we are going to use the following choices:

Mo karolong e o tla beng o bua gore thuso e botlhokwa jang, re tlile go dirisa tse di latelang:

1 2 3

Very Important

Go botlhokwa thata

Important

Go botlhokwa

Not Important

Ga go botlhokwa

*(after each question, re-read the answer choices if needed.)*

1. My parents show me they are proud of me.

Batsadi bame ba mpontsha gore ba ikgantsha ka nna.

1. My parents understand me.

Batsadi bame ba a ntlhaloganya.

1. My parents listen to me when I need to talk.

Batsadi bame ba nteetsa fa ke batla ko bua.

1. My parents give me ideas when I don’t know what to do.

Batsadi bame ba mpha megopolo fa ke saitse gore nka dirang.

1. My parents give me good advice.

Batsadi bame ba mpha kgakololo e e siameng.

1. My parents help me solve problems by giving me good information.

Batsadi bame ba nthusa go rarabolola mathata ka go mpha kitso e e siameng.

1. My parents tell me I did a good job when I do something well.

Batsadi bame ba mpolelela gore ke dirile tiro e ntle ga ke dirile sengwe sentle.

1. My parents nicely tell me when I make mistakes.

Batsadi bame ba mpolelela sentle fa ke dira diphoso.

1. My parents reward me when I’ve done something well.

Batsadi bame ba a nteboga fa ke dirile sengwe sentle.

1. My parents take time to help me decide things.

Batsadi bame ba tsaya nako go nthusa go tsaya tshwetso mo dilong.

1. My parents get me many of the things I need.

Batsadi bame ba nneela dilo ka bontsi mo go tse ke di tlhokang.

Is there anyone else in your life who helps you as much as your parents? (yes or no)

A go nale mongwe gape mo botshelong ja gago yo o go thusang fela thata jaaka batsadi ba gago? (Ee kana Nnyaa)

(If yes, continue to the section below)

Who helps you as much as your parents? (free text answer. Enter description of person, not name. (For example, “maternal aunt” not “aunt Dimpho.”)

Please answer the following questions about the person or people who help you as much as your parents. Just like with the parent questions, I am going to ask you about different kinds of help that you get from a person who helps you as much as your parents, answer the questions based on the person who helps you the most. For each kind of help, I want you to tell me two things. First, tell me how often you receive the kind of help described. Then, rate how important you think that help is to you. For the part when you tell me how often you get that type of help, we are going to use the following choices: Never. Almost never. Most of the time. Almost always. Always.

For the part where you say how important the help is, we are going to use the following choices: Not important. Important. Very important.

(Ask questions with identity of person discussed above. For example, "your aunt shows you that she is proud of you." After each question, re-read the answer choices if needed.)

Tsweetswee araba dipotso tse di latelang ka motho kana batho ba ba go thusang fela thata jaaka batsadi ba gago.

Jaaka dipotso tsa batsadi , ke tlile go go botsa ka mefuta e e farologanyeng ya thuso e o e amogelang go tswa mo mothong yo o go thusang fela thata jaaka batsadi ba gago.Fa go nale batho ba feta bongwe ba ba go thusang fela thata jaaka batsadi ba gago,araba potso o remeletse mo mothong yo o go neelang thuso go feta. Mo potsong nngwe le nngwe, ke batla o mpolelela dilo dile pedi. Lantlha, mpolelela gore o amogela thuso e e tlhalositsweng ga kae. O bo, o mpolelela gore thuso eo e botlhokwa gole ka nakang mo go wena.

Mo karolong e o mpolelelang gore o bona mofuta wa thuso e o ga kae re tlile go dirisa tse di latelang: Gotlhelele ganke. Ganke. Nako tse dingwe. Gotlehelele nako tsotlhe. Nako tsotlhe.

Mo karolonge o tla a beng o bua gore thuso e botlhokwa jang,re tlile go dirisa tse di latelang: Ga go botlhokwa, Go botlhokwa, Go botlhokwa thata.

(Botsa potsa o bua ka motho yo go builweng ka ene fa godimo. Sekai, "mmangwaneago o go bontsha gore o ikgantsha ka wena." Morago ga potso nngwe le nngwe, boelela dikarabo fa go tlhokega.)

1. ____ shows me that he/she is proud of me.

mpontsha gore o ikgantsha ka nna.

1. ____ understands me.

o a ntlhaloganya.

1. ____ listens to me when I need to talk.

o a nteetsa fa ke batla go bua.

1. ____ gives me ideas when I don’t know what to do.

o mpha megopolo fa ke saitse gore ke ka dira eng.

1. ____ gives me good advice.

o mpha kgagololo e e siameng.

1. ____ helps me solve problems by giving me good information.

o nthusa go rarabolola mathata ka go mpha kitso e e siameng.

1. ____ tells me I did a good job when I do something well.

o mpolelela gore ke dirile tiro e ntle fa ke dira sengwe sentle.

1. ____ nicely tells me when I make mistakes.

o mpolelela sentle fa ke dira diphoso.

1. ____ rewards me when I’ve done something well.

o a nteboga fa ke dirile sengwe sentle.

1. ____ takes time to help me decide things.

o tsaya naka go nthusa go tsaya tshwetso mo dilong.

1. ____ gets me many of the things I need.

o neela dilo ka bontsi tse ke di tlhokang.
